# The Serial Mediation of the Association between Breakfast Skipping and Suicidality by Weight Status and Depressive Symptoms: Findings from the National Youth Risk Behavior Surveys of the United States

**DOI:** 10.3390/nu14050956

**Published:** 2022-02-23

**Authors:** Bao-Peng Liu, Hui-Juan Fang, Cun-Xian Jia

**Affiliations:** 1 Department of Epidemiology, School of Public Health, Cheeloo College of Medicine, Shandong University, Jinan 250012, China; liubaopeng@sdu.edu.cn; 2 Center for Suicide Prevention and Research, Shandong University, Jinan 250012, China; 3 Department of Pediatrics, Shandong Provincial Hospital Affiliated to Shandong First Medical University, Jinan 250021, China; fhj000@126.com

**Keywords:** breakfast skipping, weight status, depressive symptoms, suicidality

## Abstract

Background: The evidence is limited for the dose–response association between breakfast skipping and suicidality. The underlying pathway from breakfast skipping to suicidality has also rarely been explored in previous studies. Methods: The data of Youth Risk Behavior Surveys (YRBSs) of the United States from 2011 to 2019 were used with a sample size of 74,074. The male: female ratio was nearly 1:1. Binary logistic regression models with complex sampling design were adopted to show the effect of breakfast skipping on weight status, depressive symptoms, and suicidality. Serial mediation was used to explore the association between breakfast skipping and suicidality by overweight/obesity and depressive symptoms. Findings: The weighted prevalence rates (95% confidence interval) of suicidal ideation, suicide plan, suicide attempt, and medically serious suicide attempt for skipping breakfast totally (0 times/week) were 25.6% (24.4–26.8%), 21.7% (20.5–22.9%), 14.2% (13.0–15.3%), and 5.3% (4.6–5.9%). Breakfast skipping was significantly associated with increased risk of suicidal ideation, suicide plan, suicide attempt, and medically serious suicide attempt. There was statistical significance for the linear dose–response association between breakfast skipping and overweight/obesity, depressive symptoms, and suicidality regardless of sex and age. A serial mediation with effect sizes between 39.68% and 51.30% for the association between breakfast skipping and suicidality by overweight/obesity and depressive symptoms was found in this study. Conclusions: This study emphasizes the hazards of breakfast skipping, which could increase the risk of suicidality among adolescents. Overweight/obesity and depressive symptoms as the mediating factors for the association between breakfast skipping and suicidality should also be with more attention.

## 1. Introduction

Suicide, which leads to more than 700,000 deaths every year, is a global imperative and public health problem [1,2]. Suicide is the fourth leading cause of death among 15–29-year-olds worldwide, and the second leading cause of death among 10–34-year-olds in the US [1,3,4]. Depression is an important predictor of subsequent mental disorders, self-harm, and suicidal behaviors [5,6,7]. The prevalence of depression is rising from around 5% in early adolescence to as high as 20% by the end of adolescence [7]. More attention should be paid to depression and suicide in consideration of the key period from adolescence to early adulthood.

Dietary behaviors are important for the physical and mental health of adolescents [8,9]. Breakfast, as an important daily meal, plays an important role in the intake of nutrients and energy by adolescents [10,11]. The prevalence of skipping breakfast—i.e., irregular breakfast consumption—is approximately between 29.2% and 57.2% in adolescents [11,12,13,14,15]. Previous studies have proven that skipping breakfast is associated with increased risk of overweight/obesity [14,15,16], lower cardiorespiratory fitness and less healthy cardiovascular profile [16], emotional and behavioral problems [17], primary dysmenorrhea [18], and intense impulsivity [19] in adolescents. Moreover, a systematic review and meta-analysis found that skipping breakfast was associated with several mental problems—such as depression (OR: 1.36, 95%CI: 1.30–1.43), stress (OR: 1.25, 95%CI: 0.96–1.53), anxiety (OR: 1.51, 95%CI: 1.25–1.77), and psychosocial distress (OR: 1.55, 95%CI: 1.47–1.62) [20]—for the population of adolescents. Another systematic review focusing on the association between diet and depression among children and adolescents also found that having breakfast was significantly associated with lower depressive symptoms [8]. However, sex- and age-specific associations between breakfast consumption and depression have rarely been reported in previous studies. It is also little known which factors could mediate the association between breakfast consumption and depression.

The evidence of the association between breakfast consumption and suicidality is limited. One study focusing on lifestyle and suicide-related behaviors in adolescents reported that skipping breakfast totally was related to suicidal ideation, suicide plans, and suicide attempts [21]; however, dose–response evidence related to breakfast consumption with suicidality is lacking, and whether the associations were affected by age and/or sex remains unknown. Another study taking advantage of the method of latent class analysis adopted breakfast consumption as a factor of healthy lifestyle to explore the association with suicidality and found that the lowest engagement in health-promoting behaviors, including skipping breakfast, was related to suicidality [22].

Although previous studies have explored the association between breakfast consumption and suicidality, the underlying pathway from breakfast skipping to suicidality has not been clearly examined. Previous studies have stated that overweight or obesity could significantly increase the risk of depression [23,24] and suicidality [25,26,27,28]. Based on this evidence, the following hypotheses can be built: (1) the association between breakfast skipping and suicidality might be mediated by weight status; (2) the association might be mediated by depressive symptoms; or (3) the association might be mediated by weight status and depressive symptoms simultaneously.

This study, using the data from the national Youth Risk Behavior Surveys (YRBSs) of the United States from 2011 to 2019, had the following aims: (1) to document the prevalence of suicidal ideation, suicide plans, suicide attempts, and medically serious suicide attempts by sex, age, weight status, different frequency of breakfast consumption, and the presence or absence of depressive symptoms; (2) to report the dose–response associations between breakfast consumption and weight status, depressive symptoms, and suicidality; (3) to explore the age- and sex-specific effects of breakfast skipping on weight status, depressive symptoms, and suicidality; and (4) to examine the mediating effect of the association between breakfast skipping and suicidality by weight status and depressive symptoms.

## 2. Methods

### 2.1. Design and Participants

The Youth Risk Behavior Surveys (YRBSs) were extracted from the Youth Risk Behavior Surveillance System (YRBSS), which was developed in 1990 by the Centers for Disease Control and Prevention (CDC) of the United States (US). The aim of this system was to monitor health-risk behaviors that contribute markedly to the leading causes of death, disability, and social problems and are often established during childhood and early adolescence. The YRBSs, which were conducted every two years, were approved by the institutional review board of the CDC, and are publicly available. The YRBSs were national school-based surveys of representative samples of 9th through 12th grade students, and employed a three-stage cluster sample design to include public and private schools in the 50 states and the District of Columbia. A self-administered computer-scannable questionnaire with anonymous and voluntary procedures and parental permission was used by students. Each record in the database was applied a weighting factor with adjustment for nonresponse and oversampling of Black and Hispanic/Latino students. More details about the YRBSs can be found on the following website: https://www.cdc.gov/healthyyouth/data/yrbs/data.htm (accessed on 1 October 2021), as well as in previous published studies about the YRBSs [29,30]. In consideration of data integrity (the data on breakfast consumption began from 2011), this study included the data of recent five surveys performed in the years of 2011, 2013, 2015, 2017, and 2019. The total sample size of this study was 74,074.

### 2.2. Covariates

#### 2.2.1. Demographic Factors

The demographic factors in this study included age, sex, race, and year of survey. Age was dichotomized into ≤16 years old and >16 years old according to the median of age distribution in this study. Race was categorized into White, Black or African American, Hispanic/Latino, and all other races. Race questions included: “Are you Hispanic or Latino?” and “What is your race?”. If participants responded “yes” to the former question, they were identified as “Hispanic/Latino”; otherwise, “White” and “Black or African American” were classified as such, and all other races (e.g., American Indian or Alaska Native, Asian, Native Hawaiian or Other Pacific Islander) were labelled as “Others”. Year of survey was used as a multinomial multilevel variable in the model in order to adjust for possible bias.

#### 2.2.2. Dietary Behaviors

Dietary behaviors in this study included vegetable, fruit, milk, and fizzy drink (soda or pop) consumption. The questions and responses can be seen in Table S1. In brief, the responses pertaining to vegetables, fruits, and fizzy drinks were dichotomized into one or more times per day and no more than one time per day, while the responses pertaining to milk consumption were dichotomized into one or more glasses per day and no more than one glass per day.

### 2.3. Independent Variables (Breakfast Consumption)

Breakfast consumption was measured by the question: *During the past 7 days, on how many days did you eat breakfast?* Response options were 0–7 days, and answers were categorized into daily, 4–6 times/week, 1–3 times/week, and none for analysis in this study.

### 2.4. Mediating Factors (Weight Status and Depressive Symptoms)

Weight status was categorized into normal or underweight, overweight, and obesity according to age- and sex-specific body mass index (BMI). The adolescents were considered overweight when BMI was at or above the 85th percentile, and obese when BMI was at or above the 95th percentile, for BMI by age and sex. The program and technical documentation for calculating and discriminating weight status can be found on the following website: https://www.cdc.gov/nccdphp/dnpao/growthcharts/resources/sas.htm (accessed on 1 October 2021), as well as in previous studies [22,31].

Depressive symptoms were measured by the question: *During the past 12 months, did you ever feel so sad or hopeless almost every day for two weeks or more in a row that you stopped doing some usual activities?* The responses for this question were yes or no. The questions and responses can be seen in Table S1.

### 2.5. Dependent Variables (Suicidality)

Dependent variables in this study were related to suicidality, i.e., suicidal ideation, suicide plan, suicide attempt, and medically serious suicide attempt. The responses for suicidal ideation and suicide plan were yes or no, and these items were measured by the questions: *During the past 12 months, did you ever (1) seriously consider attempting suicide? (2) make a plan about how you would attempt suicide?* Responses were dichotomized into yes and no. Suicide attempt was measured by the question: *During the past 12 months, how many times did you actually attempt suicide?* Responses were dichotomized into 0 and 1+ times. Medically serious suicide attempt was measured by the question: *If you attempted suicide during the past 12 months, did any attempt result in an injury, poisoning, or overdose that had to be treated by a doctor or nurse?* Responses were dichotomized into yes and no. The questions and responses can be seen in Table S1.

### 2.6. Statistical Analysis

All the analyses were performed using R software version 4.1.0. Codes related to complex sampling design were used to get valid point estimates, standard errors, confidence intervals, and tests of hypotheses. Weighted prevalence rates of suicidal ideation, suicide plan, suicide attempt, and medically serious suicide attempt were reported in this study. The confidence intervals of weighted prevalence were estimated by the methods proposed by Korn and Graubard [32]. Pearson’s chi-squared statistics with the second-order correction of the Rao–Scott chi-squared test [33] were used to explore the differences in weighted prevalence rates related to suicidal ideation, suicide plan, suicide attempt, and medically serious suicide attempt by age, sex, weight status, breakfast consumption, and depressive symptoms. The *p*-values were computed with a Satterthwaite approximation to the distribution and with denominator degrees of freedom, as recommended by Thomas and Rao [34]. Binary logistic regression models with complex sampling design were used to explore the associations between breakfast consumption, weight status, depressive symptoms, and suicidality. Simultaneously, age- and sex-specific effects of breakfast skipping on weight status, depressive symptoms, and suicidality were also reported. Moreover, *p*-values for linear trends were calculated by weighted binary logistic regression models considering dose–response associations between breakfast consumption, weight status, depressive symptoms, and suicidality.

The serial mediation of the association between breakfast skipping and suicidality by weight status and depressive symptoms was examined using the “bruceR” package in R. Bootstrap methods were used to verify the indirect effect and to produce confidence intervals, which were based on 1000 bootstrapping samples. Standardized coefficients were used to show the estimates of each pathway associated with indirect, direct, and total effects.

Sensitivity analysis of missing data via multiple imputation by chained equations (MICE) was used to explore the stability of the association between breakfast consumption and depressive symptoms and suicidality [29,35]. All reported probabilities (*p*-values) were two-sided, and any less than 0.05 was considered statistically significant.

## 3. Results

### 3.1. Characteristics of Included Participants

The number of included adolescents for the surveys of 2011, 2013, 2015, 2017, and 2019 was 15,425, 13,583, 15,624, 14,765, and 13,677, respectively. Included participants were mostly aged 12–18 years and had a male: female ratio of nearly 1:1. Nearly 15% and 13% of included adolescents were overweight and obese, respectively. The proportion of daily, 4–6 days/week, 1–3 days/week, and none for frequency of breakfast consumption was 29.9%, 18.5%, 24.7%, and 12.7%, respectively. Despite data from different survey years, the distribution of demographic factors was similar. More details of the distribution of age, sex, weight status, breakfast consumption, and unweighted proportions of dietary behaviors, depressive symptoms, and suicidality can be seen in Table S2. Younger, male, and normal or underweight adolescents seemed to have a higher prevalence of daily breakfast consumption. More details can be seen in Table S3.

### 3.2. Weighted Prevalence of Suicidality

As shown in Table 1, the total weighted prevalence rates of suicidal ideation, suicide plan, suicide attempt, and medically serious suicide attempt were 17.3% (16.8–17.8%), 14.0% (13.5–14.5%), 8.1% (7.7–8.5%), and 2.6% (2.4–2.8%), respectively. Younger adolescents (≤16 years old) had higher prevalence rates of suicidal ideation (*p* = 0.035), suicide plan (*p* = 0.016), and suicide attempt (*p* < 0.001). Although younger adolescents had a higher prevalence of medically serious suicide attempts, the difference did not appear to be statistically significant (*p* = 0.057). The girls had higher prevalence rates of suicidal ideation, suicide plan, suicide attempt, and medically serious suicide attempt than the boys (all *p* < 0.001).

As the frequency of breakfast consumption decreased, the prevalence of suicidal ideation, suicide plan, suicide attempt, and medically serious suicide attempt increased. The weighted prevalence rates of suicidal ideation, suicide plan, suicide attempt, and medically serious suicide attempt for skipping breakfast totally (0 times/week) were 25.6% (24.4–26.8%), 21.7% (20.5–22.9%), 14.2% (13.0–15.3%), and 5.3% (4.6–5.9%), respectively. The prevalence rates of suicidal ideation, suicide plan, suicide attempt, and medically serious suicide attempt among adolescents with depressive symptoms were much higher than among those without.

### 3.3. The Effect of Breakfast Consumption on Weight Status, Depressive Symptoms, and Suicidality

Table 2 reports the associations between breakfast consumption, weight status, depressive symptoms, and suicidality. Model 1, adjusting for age, sex, race, survey year, and dietary behaviors—including vegetable, fruit, milk, and fizzy drink consumption—showed that compared with breakfast consumption daily, 4–6 days per week (OR: 1.19, 95%CI: 1.10–1.28), 1–3 days per week (OR: 1.43, 95%CI: 1.33–1.54), and none (OR: 1.60, 95%CI: 1.46–1.75) were associated with increased risk of overweight and obesity. Similarly, skipping breakfast—regardless of frequency—was related to depressive symptoms, suicidal ideation, suicide plan, and suicide attempt. However, only breakfast consumption less than 3 days per week was significantly associated with medically serious suicide attempt. Furthermore, there was statistical significance for the linear dose–response association between breakfast skipping and depressive symptoms, suicidal ideation, suicide plan, suicide attempt, and medically serious suicide attempt (all *p* for trend < 0.001). Skipping breakfast totally could increase the risk of depressive symptoms or suicidality in adolescents by 2–3-fold.

Model 2, which added weight status to Model 1, reported similar results. Breakfast skipping was statistically associated with increased risk of depressive symptoms, suicidal ideation, suicide plan, and suicide attempt. Only breakfast consumption less than 3 days per week had a statistically significant association with medically serious suicide attempt. Overweight or obesity could also increase the risk of depressive symptoms (OR: 1.18, 95%CI: 1.12–1.25), suicidal ideation (OR: 1.36, 95%CI: 1.27–1.45), suicide plan (OR: 1.33, 95%CI: 1.24–1.43), suicide attempt (OR: 1.31, 95%CI: 1.19–1.45), and medically serious suicide attempt (OR: 1.40, 95%CI: 1.19–1.64). Model 3, which added depressive symptoms to Model 1, reported that breakfast skipping was statistically associated with increased risk of suicidal ideation and suicide plan. Only breakfast consumption less than 3 days per week was significantly associated with suicide attempt and medically serious suicide attempt. Depressive symptoms could increase the risk of suicidality 10-fold, including suicidal ideation, suicide plan, suicide attempt, and medically serious suicide attempt. Similar results could also be seen in the full model (Model 4), which added weight status and depressive symptoms simultaneously to Model 1.

**Figure 1:**
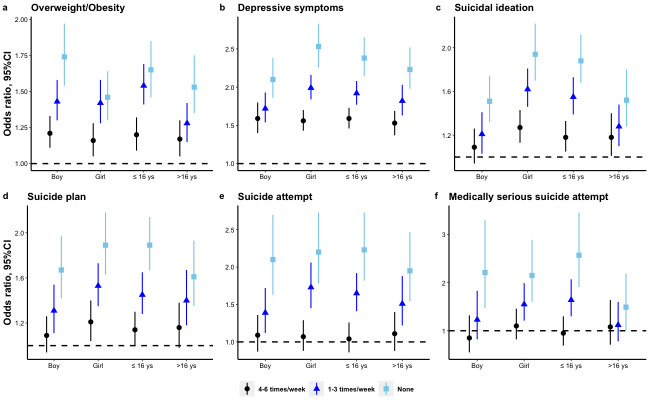
The age- and sex-specific effects of breakfast skipping on weight status, depressive symptoms, and suicidality among high school students in the US: (**a**) overweight/obesity, (**b**) depressive symptoms, (**c**) suicidal ideation, (**d**) suicide plan, (**e**) suicide attempt, (**f**) medically serious suicide attempt. The estimates of overweight/obesity were adjusted for age, sex, race, survey year, and dietary behaviors, including vegetable, fruit, milk, and fizzy drink consumption. The estimates of depressive symptoms were adjusted for age, sex, race, survey year, weight status, and dietary behaviors, including vegetable, fruit, milk, and fizzy drink consumption. The estimates of suicidality were adjusted for age, sex, race, survey year, weight status, depressive symptoms, and dietary behaviors including vegetable, fruit, milk, and fizzy drink consumption. CI: confidence interval.

Sex- and age-specific associations between breakfast consumption and weight status, depressive symptoms, and suicidality were similar to the total estimates; the details can be seen in Figure 1. The interactive effects of breakfast skipping and age or sex on the relevant outcomes can be seen in Table S4. Specifically, the effect of breakfast skipping among young adolescents (≤16 years old) on overweight/obesity, suicidal ideation, and medically serious suicide attempt was significantly greater compared with elder adolescents. Female adolescents who skipped breakfast had a slightly greater effect in terms of depressive symptoms and suicidal ideation.

### 3.4. Serial Mediation

**Figure 2:**
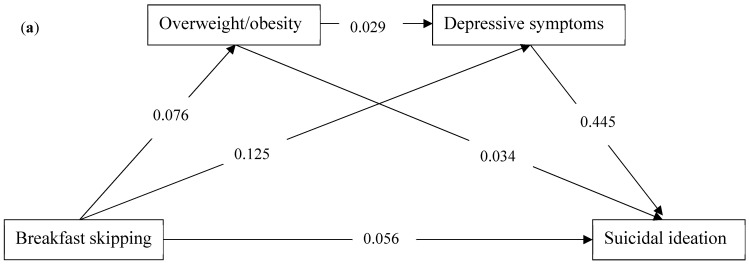

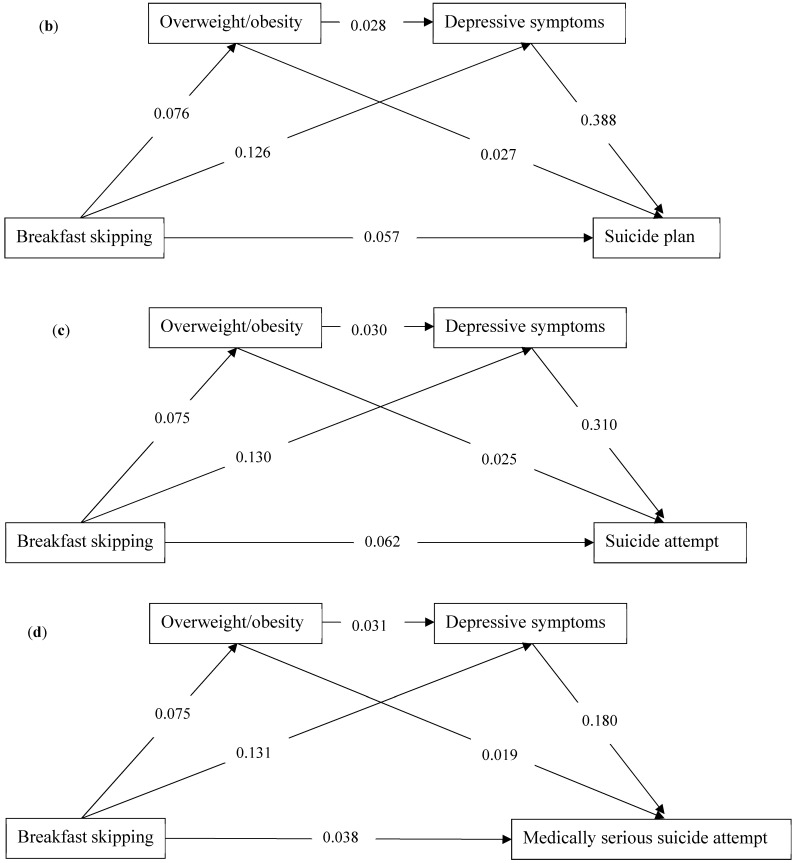
The serial mediating effect of the associations between breakfast skipping and suicidality by weight status and depressive symptoms among high school students in the US: (**a**) suicidal ideation, (**b**) suicide plan, (**c**) suicide attempt, (**d**) medically serious suicide attempt. The numbers on the paths represent standardized coefficients.

The serial mediation analysis models are illustrated in Figure 2a–d. Four models were built among breakfast skipping, overweight/obesity, depressive symptoms, and suicidality, including suicidal ideation (Figure 2a), suicide plan (Figure 2b), suicide attempt (Figure 2c), and medically serious suicide attempt (Figure 2d). Breakfast skipping was positively associated with overweight/obesity, depressive symptoms, and suicidality. Overweight/obesity was positively associated with depressive symptoms and suicidality. Each path was statistically significant for the association between breakfast skipping, overweight/obesity, depressive symptoms, and suicidality. The total indirect effect for the association with suicidal ideation, suicide plan, suicide attempt, and medically serious suicide attempt was 0.059 (0.055, 0.063), 0.052 (0.048, 0.055), 0.043 (0.040, 0.046), and 0.025 (0.023, 0.028), respectively. The direct effect for the association between breakfast skipping and suicidal ideation, suicide plan, suicide attempt, and medically serious suicide attempt was 0.056 (0.048, 0.064), 0.057 (0.048, 0.065), 0.062 (0.053, 0.072), and 0.038 (0.028, 0.048), respectively. The effect sizes for the mediations with suicidal ideation, suicide plan, suicide attempt, and medically serious suicide attempt were 51.30%, 47.71%, 40.95%, and 39.68%, respectively. The mediations were mostly from the pathway between breakfast skipping, depressive symptoms and suicidality. More details of serial mediation can be seen in Table 3.

### 3.5. Sensitivity Analysis

Multiple imputation by chained equations (MICE) was performed to explore the effect of missing data on the association between breakfast skipping and suicidality. Sensitivity analyses by MICE found that the odds of suicidality were slightly changed, and revealed that the estimates were stable.

## 4. Discussions

### 4.1. Main Findings

This study used the survey data of YRBSs from 2011 to 2019, firstly examining the association between breakfast consumption and suicidality among adolescents. The main findings were as follows: (1) The weighted prevalence of suicidal ideation, suicide plan, suicide attempt, and medically serious suicide attempt for skipping breakfast totally (0 times/week) was 25.6% (24.4–26.8%), 21.7% (20.5–22.9%), 14.2% (13.0–15.3%), and 5.3% (4.6–5.9%), respectively. (2) There was statistical significance for the linear dose–response association between breakfast skipping and overweight/obesity, depressive symptoms, and suicidality. (3) A serial mediation for the association between breakfast skipping and suicidality by overweight/obesity and depressive symptoms was found in this study.

### 4.2. The Effect of Breakfast Skipping on Suicidality

The proportion of breakfast skipping, regardless of the frequency, was nearly 70% in this study, which is higher than the estimates reporting a range between 29.2% and 57.2% from previous studies [11,12,13,14,15]. The weighted prevalence of the past year of suicidal ideation, suicide plan, and suicide attempt in this study was higher than that reported in China [36], and comparable with some other countries [37]. Consistent with previous studies [36,37], a higher prevalence of suicidality among female adolescents was also found in this study.

Li et al. [21], taking advantage of the data from the YRBS in 2019, found that skipping breakfast totally was associated with increased risk of suicidal ideation (OR:1.39, 95%CI: 1.41–1.71), suicide plan (OR: 1.49, 95%CI: 1.12–1.98), and suicide attempt (OR: 1.64, 95%CI: 1.18–2.27). In this study, we used the data of the YRBSs from 2011 to 2019 and found that skipping breakfast was also related to medically serious suicide attempt. Moreover, this study adds some evidence for the linear dose–response association between breakfast skipping and suicidality. In the full model, skipping breakfast occasionally could increase the risk of suicidality by 1.1–1.6-fold, while skipping breakfast totally could increase the risk of suicidality by 1.7–2.1-fold. It is possible that intervention for breakfast skipping is necessary in the education of dietary behaviors among adolescents. Although previous studies [21,22] have tended to summarize breakfast consumption into lifestyle behaviors in order to explore the association with suicidality, breakfast consumption itself—as an indispensable meal and a source of nutrients—might play an important role in nutritious factors, and therefore should not be ignored.

Although this study found a significant association between skipping breakfast and suicidality, the intensity of association varied by age and sex. Breakfast skipping among female adolescents seemed to have a higher effect on suicidal ideation compared with male adolescents. This could be explained by the following reasons: Firstly, an Italian study found that skipping breakfast was only associated with low fruit and vegetable intake among female adolescents [38], which was reported to be related to increased risk of depressive symptoms and suicidality [39,40]. Secondly, daily breakfast consumption was often accompanied by other healthy lifestyles, such as suitable time of physical activity, among male adolescents [15], which has been reported to have a negative association with suicidality [41]. Although this study and previous studies found that breakfast skipping was more frequent in older adolescents [39,42], the effect of regular breakfast consumption on suicidality seemed to be greater among younger adolescents in this study. This could be explained by the fragility of younger adolescents at the beginning of rapid growth and development associated with nutrient intake, which could have an effect on mental health. More longitudinal studies and clinical trials are needed in order to prove the differences in the associations related to age and sex.

### 4.3. The Effect of Breakfast Skipping on Overweight/Obesity and Depressive Symptoms

Consistent with previous studies, we also found that breakfast skipping was associated with overweight/obesity [14,15,16] and depressive symptoms [20] in the adolescents, regardless of age and sex. The risk of depressive symptoms associated with breakfast skipping was slightly higher than the pooled results of the meta-analysis [20]. A previous meta-analysis and systematic review of observational studies found that there were no well-defined mechanisms between breakfast skipping and depressive symptoms and provided a possibility for the association caused by overweight or obesity [20]. This study provides effective evidence of the mediating effect of overweight/obesity on the association between skipping breakfast and depressive symptoms. More longitudinal studies are needed in order to prove the causality of the association and the pathway from breakfast skipping to mental health issues, such as depression, anxiety, and stress.

### 4.4. Serial Mediations from Breakfast Skipping to Suicidality

It is worth noting that this study found serial mediations by overweight/obesity and depressive symptoms on the associations between breakfast skipping and suicidality, including suicidal ideation, suicide plan, suicide attempt, and medically serious suicide attempt. This is the first study to explore the pathway underlying the association between breakfast skipping and suicidality. Although the effects of the pathway for breakfast skipping, overweight/obesity, depressive symptoms, and suicidality were relatively low, the findings in this study provide clear evidence for the association. Apart from depressive symptoms, many other important mental factors, such as anxiety, stress, and psychosocial distress [20]—which could be affected by breakfast skipping—might have effect on suicidality among adolescents. Moreover, physical unfitness—including CVD risk factors such as cardiorespiratory fitness and cardiovascular profile [16]—primary dysmenorrhea [18], emotional and behavioral problems [17], and personality issues such as intense impulsivity [19] might also be mediating factors between breakfast skipping and suicidality. Considering that causality is not as certain in this study, it is possible that various factors—including suicidality, depressive symptoms, and overweight or obesity—could lead to breakfast skipping. More studies are needed to explore other paths for the association, and longitudinal studies are also needed in order to determine the causal association in the future.

### 4.5. Strengths and Limitations

This study has several strengths. Firstly, this study used a national sample of US adolescents, which included five surveys from 2011 to 2019, with good representation. Secondly, this study found a linear dose–response relationship between breakfast skipping and suicidality. Thirdly, this study explored the underlying pathway and mediating effects by weight status and depressive symptoms for the association between breakfast skipping and suicidality.

Some limitations should be considered with caution when extrapolating the conclusions. First, this study only explored the effect of the frequency of breakfast consumption on weight status, depressive symptoms, and suicidality. However, the quantity and quality of breakfast consumption are hard to include for consideration. The effects caused by other meals, such as lunch and dinner, were also not able to be explored because of limited data. Second, the causal relationships between breakfast consumption, weight status, depressive symptoms, and suicidality could not be proven in this study, because of its cross-sectional design. Third, although the sample size was relatively large, it is unclear whether this study could be generalized to adolescents in other regions—especially those in developing countries. In addition, breakfast skipping might be affected by the family environment [43], which could also affect mental problems and suicidality. More studies are needed to explore these relationships. Finally, all of the data were reported by the adolescents themselves; thus, recall bias might affect the conclusions.

## 5. Conclusions

There is a linear dose–response relationship between breakfast skipping and increased risk of suicidality among adolescents, regardless of age and sex. Breakfast skipping could increase the risk of suicidality via the mediation of overweight/obesity and depressive symptoms. These findings highlight the importance of breakfast consumption, and relevant departments and schools should formulate corresponding measures to ensure that adolescents eat breakfast regularly.

## Figures and Tables

**Table 1:** The weighted prevalence of suicidality among high school students in the US.

Variables	Weighted Prevalence, % (95%CI)			
	Suicidal Ideation	Suicide Plan	Suicide Attempt	Medically Serious Suicide Attempt
Total	17.3 (16.8–17.8)	14.0 (13.5–14.5)	8.1 (7.7–8.5)	2.6 (2.4–2.8)
Age (years old)				
≤16	17.6 (17.0–18.1)	14.3 (13.7–14.9)	8.8 (8.3–9.2)	2.7 (2.4–2.9)
>16	16.8 (16.1–17.4)	13.5 (12.9–14.1)	7.0 (6.5–7.6)	2.4 (2.1–2.6)
*p*-Value for differences ^a^	0.035	0.016	<0.001	0.057
Sex				
Boy	12.3 (11.8–12.8)	10.4 (9.9–10.8)	5.7 (5.2–6.1)	1.8 (1.5–2.0)
Girl	22.2 (21.4–23.1)	17.6 (16.8–18.4)	10.4 (9.8–11.1)	3.3 (3.0–3.6)
*p*-Value for differences ^a^	<0.001	<0.001	<0.001	<0.001
Weight status				
Normal or lower weight	15.7 (15.2–16.3)	12.7 (12.1–13.2)	7.1 (6.6–7.5)	2.1 (1.9–2.3)
Overweight/obesity	20.2 (19.3–21.1)	16.3 (15.5–17.1)	9.4 (8.8–10.1)	3.0 (2.7–3.4)
*p*-Value for differences ^a^	<0.001	<0.001	<0.001	<0.001
Breakfast consumption				
Daily	11.5 (11.0–12.0)	9.3 (8.8–9.9)	4.7 (4.3–5.1)	1.5 (1.3–1.9)
4–6 days/week	16.9 (16.0–17.8)	13.2 (12.4–14.0)	6.6 (6.0–7.2)	1.8 (1.5–2.1)
1–3 days/week	21.0 (20.1–21.9)	16.9 (16.1–17.8)	10.1 (9.5–10.8)	3.0 (2.7–3.3)
None	25.6 (24.4–26.8)	21.7 (20.5–22.9)	14.2 (13.0–15.3)	5.3 (4.6–5.9)
*p*-Value for differences ^a^	<0.001	<0.001	<0.001	<0.001
Depressive symptoms				
Yes	43.6 (42.6–44.7)	34.7 (33.7–35.6)	21.0 (20.1–21.8)	6.9 (6.3–7.4)
No	5.3 (5.0–5.6)	4.6 (4.4–4.9)	2.1 (1.9–2.4)	0.5 (0.4–0.6)
*p*-Value for differences ^a^	<0.001	<0.001	<0.001	<0.001

^a^: Pearson’s chi-squared statistics with the second-order correction of the Rao–Scott chi-squared test were used, and the *p*-values were computed with a Satterthwaite approximation to the distribution and with denominator degrees of freedom, as recommended by Thomas and Rao.

**Table 2:** The associations between breakfast consumption, weight status, depressive symptoms, and suicidality among high school students in the US.

Variables	OR (95%CI)					
	Overweight/Obesity	Depressive Symptoms	Suicidal Ideation	Suicide Plan	Suicide Attempt	Medically Serious Suicide Attempt
Model 1 ^a^						
Breakfast consumption ^b^						
Daily	Reference	Reference	Reference	Reference	Reference	Reference
4–6 days/week	1.19 (1.10–1.28) ***	1.56 (1.46–1.67) ***	1.47 (1.36–1.58) ***	1.40 (1.28–1.53) ***	1.32 (1.16–1.50) ***	1.21 (0.97–1.51)
1–3 days/week	1.43 (1.33–1.54) ***	1.89 (1.78–2.01) ***	1.91 (1.78–2.06) ***	1.88 (1.73–2.06) ***	2.16 (1.94–2.40) ***	1.94 (1.62–2.32) ***
None	1.60 (1.46–1.75) ***	2.34 (2.16–2.54) ***	2.52 (2.31–2.75) ***	2.58 (2.33–2.85) ***	3.19 (2.77–3.68) ***	3.49 (2.82–4.32) ***
Model 2 ^c^						
Breakfast consumption ^b^						
Daily		Reference	Reference	Reference	Reference	Reference
4–6 days/week		1.57 (1.47–1.68) ***	1.47 (1.36–1.59) ***	1.42 (1.29–1.56) ***	1.35 (1.18–1.54) ***	1.25 (0.99–1.59)
1–3 days/week		1.89 (1.77–2.02) ***	1.91 (1.77–2.07) ***	1.89 (1.72–2.08) ***	2.17 (1.94–2.43) ***	2.00 (1.64–2.43) ***
None		2.34 (2.15–2.55) ***	2.48 (2.26–2.71) ***	2.53 (2.28–2.82) ***	3.14 (2.70–3.66) ***	3.29 (2.60–4.16) ***
Weight status ^c^						
Normal or underweight		Reference	Reference	Reference	Reference	Reference
Overweight/obesity		1.18 (1.12–1.25) ***	1.36 (1.27–1.45) ***	1.33 (1.24–1.43) ***	1.31 (1.19–1.45) ***	1.40 (1.19–1.64) **
Model 3 ^d^						
Breakfast consumption ^b^						
Daily			Reference	Reference	Reference	Reference
4–6 days/week			1.20 (1.09–1.31) ***	1.14 (1.04–1.26) **	1.06 (0.92–1.21)	0.98 (0.79–1.23)
1–3 days/week			1.45 (1.34–1.57) ***	1.43 (1.31–1.57) ***	1.61 (1.44–1.81) ***	1.41 (1.17–1.70) ***
None			1.78 (1.62–1.96) ***	1.83 (1.65–2.03) ***	2.20 (1.89–2.55) ***	2.31 (1.86–2.88) ***
Depressive symptoms						
No			Reference	Reference	Reference	Reference
Yes			12.73 (11.72–13.82) ***	10.25 (9.46–11.10) ***	12.32 (10.78–14.08) ***	14.70 (11.37–19.00) ***
Model 4 ^e^						
Breakfast consumption ^b^						
Daily			Reference	Reference	Reference	Reference
4–6 days/week			1.19 (1.09–1.31) ***	1.16 (1.04–1.28) **	1.07 (0.93–1.23)	1.00 (0.79–1.27)
1–3 days/week			1.45 (1.33–1.58) ***	1.45 (1.31–1.59) ***	1.61 (1.43–1.82) ***	1.43 (1.17–1.75) ***
None			1.75 (1.59–1.93) ***	1.80 (1.61–2.00) ***	2.16 (1.84–2.53) ***	2.15 (1.69–2.73) ***
Weight status						
Normal or underweight			Reference	Reference	Reference	Reference
Overweight/obesity			1.31 (1.21–1.41) ***	1.27 (1.17–1.37) ***	1.24 (1.12–1.37) ***	1.31 (1.11–1.55) **
Depressive symptoms						
No			Reference	Reference	Reference	Reference
Yes			12.58 (11.54–13.72) ***	10.01 (9.19–10.90) ***	12.51 (10.83–14.46) ***	15.99 (12.21–20.95) ***

OR: odds ratio; CI: confidence interval; ***: *p* < 0.001; **: *p* < 0.01; ^a^: Model 1 included the variables age, sex, race, survey year, dietary behaviors—including vegetable, fruit, milk, and fizzy drink consumption—and breakfast consumption. ^b^: *p*-Values for trend were all statistically significant in all of the models. c: Model 2 included the variables in Model 1, as well as weight status. ^d^: Model 3 included the variables in Model 1, as well as depressive symptoms. ^e^: Model 4 included the variables in Model 1, as well as weight status and depressive symptoms.

**Table 3:** The serial mediation of breakfast consumption and suicidality by weight status and depressive symptoms among high school students in the US.

Pathway	Estimate ^a^	Z	*p* Value	95%CI for the Estimate ^b^	Effect Size (%)
Suicidal ideation					51.30
Total indirect effect	0.059	27.79	<0.001	0.055, 0.063	
X-M1-Y1	0.003	7.77	<0.001	0.002, 0.003	
X-M2-Y1	0.056	26.56	<0.001	0.052, 0.060	
X-M1-M2-Y1	0.001	6.03	<0.001	0.001, 0.001	
Direct effect (X-Y1)	0.056	13.84	<0.001	0.048, 0.064	
Total effect	0.115	26.20	<0.001	0.107, 0.124	
Suicide plan					47.71
Total indirect effect	0.052	27.62	<0.001	0.048, 0.055	
X-M1-Y2	0.002	5.97	<0.001	0.001, 0.003	
X-M2-Y2	0.049	26.44	<0.001	0.045, 0.053	
X-M1-M2-Y2	0.001	6.16	<0.001	0.001, 0.001	
Direct effect (X-Y2)	0.057	13.05	<0.001	0.048, 0.065	
Total effect	0.109	23.02	<0.001	0.099, 0.118	
Suicide attempt					40.95
Total indirect effect	0.043	25.45	<0.001	0.040, 0.046	
X-M1-Y3	0.002	5.08	<0.001	0.001, 0.003	
X-M2-Y3	0.040	24.45	<0.001	0.037, 0.044	
X-M1-M2-Y3	0.001	6.22	<0.001	0.001, 0.001	
Direct effect (X-Y3)	0.062	13.14	<0.001	0.053, 0.072	
Total effect	0.105	21.56	<0.001	0.095, 0.115	
Medically serious suicide attempt					39.68
Total indirect effect	0.025	19.50	<0.001	0.023, 0.028	
X-M1-Y3	0.001	3.59	<0.001	0.001, 0.002	
X-M2-Y3	0.024	19.50	<0.001	0.021, 0.026	
X-M1-M2-Y3	<0.001	5.95	<0.001	0.000, 0.001	
Direct effect (X-Y3)	0.038	7.28	<0.001	0.028, 0.048	
Total effect	0.063	11.81	<0.001	0.053, 0.074	

X: breakfast skipping; M1: overweight/obesity; M2: depressive symptoms; Y: suicidality (Y1: suicidal ideation; Y2: suicide plan; Y3: suicide attempt; Y4: medically serious suicide attempt). a: The estimates of all the pathways were from standardized coefficients of respective models. b: 95% confidence intervals of indirect effects were estimated by percentile bootstrap with 1000 simulation samples.

## Data Availability

The data can be downloaded from https://www.cdc.gov/healthyyouth/data/yrbs/data.htm/ (accessed on 1 October 2021).

## Supplementary Materials

The following supporting information can be downloaded at https://www.mdpi.com/article/10.3390/nu14050956/s1: Table S1: Question wording and details for included variables; Table S2: Characteristics of included variables among Youth Risk Behavior Surveys by survey year (2011–2019); Table S3: The distribution of age, sex, and weight status by breakfast consumption among the US adolescents; Table S4: Interactive effects of breakfast skipping, age, and sex on weight status, depressive symptoms, and suicidality.

## Abbreviations

OR: odds ratio; CI: confidence interval; YRBS: Youth Risk Behavior Survey; BMI: body mass index.
