# Supplementary material for: The Serial Mediation of the Association between Breakfast Skipping and Suicidality by Weight Status and Depressive Symptoms: Findings from the National Youth Risk Behavior Surveys of the United States
Source: Nutrients. 2022 Feb 23;14(5):956. doi: 10.3390/nu14050956 (PMC8912887; doi:10.3390/nu14050956)
Supplement: Supplementary file 1 [file nutrients-14-00956-s001.zip › supplementary material-updated by author.pdf]

Table S1: Question wording and details for included variables

Variables	Question	Response options	Coding for analysis
Breakfast consumption	*During the past 7 days, on how many days did you eat breakfast?*	0 days, 1 day, 2 days, 3 days, 4 days, 5 days, 6 days, 7 days	Responses were categorized into none, 1-3 times/days, 4-6 times/days, and daily
Vegetable consumption	*During the past 7 days, how many times did you eat (1) green salad? (2) potatoes (Do not count French fries, fried potatoes, or potato chips? (3) carrots? (4) other vegetables? (Do not count green salad, potatoes, or carrots.)*	I did not eat… during the past 7 days, 1 to 3 times during the past 7 days, 4 to 6 times during the past 7 days, 1 time per day, 2 times per day, 3 times per day, 4 or more times per day	After summing the frequency of the four questions, responses were dichotomized into one or more times per day and no more than one time per day
Fruit consumption	*During the past 7 days, how many times did you (1) drink 100% fruit juices such as orange juice, apple juice, or grape juice? (Do not count punch, Kool-Aid, sports drinks, or other fruit-flavored drinks.) (2) eat fruit? (Do not count fruit juice)*	I did not eat/drink… during the past 7 days, 1 to 3 times during the past 7 days, 4 to 6 times during the past 7 days, 1 time per day, 2 times per day, 3 times per day, 4 or more times per day	After summing the frequency of the two questions, responses were dichotomized into one or more times per day and no more than one time per day
Milk consumption	*During the past 7 days, how many glasses of milk did you drink? (Count the milk you drank in a glass or cup, from a carton, or with cereal. Count the half pint of milk served at school as equal to one glass)*	I did not drink milk during the past 7 days, 1 to 3 glasses during the past 7 days, 4 to 6 glasses during the past 7 days, 1 glass per day, 2 glasses per day, 3 glasses per day, 4 or more glasses per day	Responses were dichotomized into one or more glasses per day and no more than one glass per day
Drinking consumption	*During the past 7 days, how many times did you drink a can, bottle, or glass of*	I did not drink soda or pop during the past 7 days, 1 to 3 times during	Responses were dichotomized into one or more times per day

	soda or pop, such as Coke, Pepsi, or Sprite? (Do not count diet soda or diet pop.)	the past 7 days, 4 to 6 times during the past 7 days, 1 time per day, 2 times per day, 3 times per day, 4 or more times per day	and no more than one time per day
Depressive symptoms	*During the past 12 months, did you ever feel so sad or hopeless almost every day for two weeks or more in a row that you stopped doing some usual activities?*	Yes or no	Responses were dichotomized into yes and no.
Suicidal ideation	*During the past 12 months, did you ever seriously consider attempting suicide?*	Yes or no	Responses were dichotomized into yes and no.
Suicide plan	*During the past 12 months, did you ever make a plan about how you would attempt suicide?*	Yes or no	Responses were dichotomized into yes and no.
Suicide attempt	*During the past 12 months, how many times did you actually attempt suicide?*	0 times, 1 time, 2 or 3 times, 4 or 5 times, 6 or more times	Responses were dichotomized into 0 and 1+times.
Medically serious suicide attempt	*If you attempted suicide during the past 12 months, did any attempt result in an injury, poisoning, or overdose that had to be treated by a doctor or nurse?*	Yes or no	Responses were dichotomized into yes and no.

Table S2: Characteristics of included variables among youth risk behavior surveys by survey year (2011-2019)

Variables	Total, n (%) [a]	Survey year, n (%)				
		2011	2013	2015	2017	2019
**Total number**	73074 (100)	15425 (21.1)	13583 (18.6)	15624 (21.4)	14765 (20.2)	13677 (18.7)
**Demographic factors**						
**Age (years old)**						
≤16	44631 (61.1)	9160 (59.4)	7713 (56.8)	9594 (61.4)	9277 (62.8)	8887 (65.0)
>16	28085 (38.4)	6203 (40.2)	5793 (42.6)	5964 (38.2)	5407 (36.6)	4718 (34.5)
**Sex**						
Boy	36108 (49.4)	7656 (49.6)	6950 (51.2)	7749 (49.6)	7112 (48.2)	6641 (48.6)
Girl	36497 (49.9)	7708 (50.0)	6621 (48.7)	7757 (49.6)	7526 (51.0)	6885 (50.3)
**Weight**						
Normal or underweight	46083 (63.1)	10069 (65.3)	8729 (64.3)	9822 (62.9)	9051 (61.3)	8412 (61.5)
Overweight/obesity	20426 (28.0)	4216 (27.3)	3851 (28.4)	4536 (29.0)	4095 (27.7)	3728 (27.3)
**Breakfast consumption**						
Daily	21883 (29.9)	4387 (28.4)	4875 (35.9)	4800 (30.7)	4135 (28.0)	3686 (27.0)
4-6 days/week	13531 (18.5)	2579 (16.7)	2735 (20.1)	3102 (19.9)	2625 (17.8)	2490 (18.2)
1-3 days/week	18037 (24.7)	3381 (21.9)	3768 (27.7)	3995 (25.6)	3432 (23.2)	3461 (24.7)
None	9254 (12.7)	1668 (10.8)	1945 (14.3)	1959 (12.5)	1726 (11.7)	1956 (12.7)
**Dietary behaviors**						
**Vegetable consumption**						
One or more times per day	40625 (55.6)	8579 (55.6)	8036 (59.2)	9043 (57.9)	8026 (54.4)	6941 (50.7)
Less than one time per day	27812 (38.1)	5862 (38.0)	5191 (38.2)	6136 (39.3)	5807 (39.3)	4816 (35.2)
**Fruit consumption**						
One or more times per day	42797 (58.6)	9229 (59.8)	8453 (62.2)	9493 (60.8)	8455 (57.3)	7167 (52.4)
Less than one time per day	27353 (37.4)	5425 (35.2)	4869 (35.8)	5808 (37.2)	5889 (39.9)	5362 (39.2)
**Milk consumption**						
One or more glasses per day	21353 (29.2)	5161 (33.5)	5081 (37.4)	4860 (31.1)	3599 (24.4)	2652 (19.4)
Less than one glass per day	38829 (53.1)	7177 (46.5)	8199 (60.4)	8355 (53.5)	8261 (55.9)	6837 (50.0)
**Soda or pop consumption**						
Less than one time per day	53237 (72.9)	10304 (66.8)	9734 (71.7)	11879 (76.0)	11654 (78.9)	9666 (70.7)
One or more times per day	15382 (21.0)	4000 (25.9)	3590 (26.4)	3413 (21.8)	2650 (17.9)	1729 (12.6)
**Depressive symptoms or suicidality**						
**Depressive symptoms**						
Yes	22969 (31.4)	4537 (29.4)	4086 (30.1)	4789 (30.7)	4631 (31.4)	4926 (36.0)
no	49198 (67.3)	10732 (69.6)	9409 (69.3)	10666 (68.3)	9896 (67.0)	8495 (62.1)
**Suicidal ideation**						
Yes	12695 (17.4)	2424 (15.7)	2259 (16.6)	2808 (18.0)	2571 (17.4)	2633 (19.3)
no	59513 (81.4)	12869 (83.4)	11232 (82.7)	12626 (80.8)	11982 (81.2)	10804 (79.0)
**Suicide plan**						
Yes	10401 (14.2)	2015 (13.1)	1874 (13.8)	2331 (14.9)	2030 (13.7)	2151 (15.7)

Variables	Total, n (%) [a]	Survey year, n (%)				
		2011	2013	2015	2017	2019
no	61466 (84.1)	13263 (86.0)	11611 (85.5)	12810 (82.0)	12511 (84.7)	11271 (82.4)
**Suicide attempt**						
Yes	5301 (7.3)	1179 (7.6)	1015 (7.5)	1203 (7.7)	837 (5.7)	1067 (7.8)
no	53968 (73.9)	12335 (80.0)	10967 (80.7)	11364 (72.7)	9849 (66.7)	9453 (69.1)
**Medically serious suicide attempt**						
Yes	1582 (2.2)	348 (2.3)	324 (2.4)	399 (2.6)	286 (1.9)	225 (1.6)
no	54827 (75.0)	12611 (81.8)	11426 (84.1)	11932 (76.4)	10334 (70.0)	8524 (62.3)

a: Missing values were existed in the reported variables.

Table S3: The distribution of age, sex, and weight status by breakfast consumption among the U.S. adolescents

Variables	Frequency of breakfast consumption, n (%)			
	**daily**	**4-6 days/week**	**1-3 days/week**	**None**
**Age (years old)**				
≤16	13654 (30.6)	8116 (18.2)	10448 (23.4)	5351 (12.0)
>16	8118 (28.9)	5354 (19.1)	7499 (26.7)	3846 (13.7)
**Sex**				
Boy	12048 (33.4)	6461 (17.9)	7952 (22.0)	4432 (12.3)
Girl	9723 (26.6)	6991 (19.2)	9985 (27.4)	4743 (13.0)
**Weight**				
Normal or underweight	14640 (31.8)	8771 (19.0)	10927 (23.7)	5202 (11.3)
Overweight/obesity	5440 (26.6)	3709 (18.2)	5556 (27.2)	2954 (14.5)

a: Missing values were existed in the reported variables.

Table S4: Interactive effect of breakfast skipping and age and sex on weight status, depressive symptoms, and suicidality

Interactive effect	Odds Ratio, 95% CI [a]					
	**Overweight/obesity**	**Depressive symptoms**	**Suicidal ideation**	**Suicide plan**	**Suicide attempt**	**Medically serious suicide attempt**
**Age**						
Age (>16 years old) * breakfast consumption (4-6 days/week)	0.98 (0.85-1.13)	0.94 (0.83-1.08)	1.00 (0.81-1.23)	1.01 (0.82-1.25)	1.04 (0.76-1.43)	1.09 (0.64-1.88)
Age (>16 years old) * breakfast consumption (1-3 days/week)	**0.84 (0.74-0.95)** **	0.92 (0.80-1.05)	**0.82 (0.68-0.98)** *	0.94 (0.76-1.17)	0.89 (0.69-1.15)	**0.66 (0.44-0.98)** *
Age (>16 years old) * breakfast consumption (None)	0.95 (0.81-1.11)	0.91 (0.79-1.05)	0.82 (0.67-1.00)	0.86 (0.70-1.05)	0.88 (0.66-1.17)	**0.56 (0.35-0.89)** *
**Sex**						
Male adolescent * breakfast consumption (4-6 days/week)	1.02 (0.90-1.16)	1.01 (0.86-1.19)	0.87 (0.72-1.05)	0.89 (0.72-1.10)	1.01 (0.75-1.37)	0.73 (0.44-1.22)
Male adolescent * breakfast consumption (1-3 days/week)	0.96 (0.83-1.10)	**0.85 (0.74-0.98)** *	**0.75 (0.62-0.91)** **	0.84 (0.68-1.03)	0.81 (0.60-1.08)	0.77 (0.49-1.22)
Male adolescent * breakfast consumption (None)	1.12 (0.96-1.30)	**0.81 (0.68-0.96)** *	**0.79 (0.65-0.95)** *	0.85 (0.69-1.06)	0.96 (0.69-1.34)	1.02 (0.61-1.70)

a: The estimates of interactive item related to overweight/obesity were adjusted for age, sex, race, survey year, and dietary behaviors including vegetable, fruit, milk, drinking consumption, and breakfast consumption. The estimates of interactive item related to depressive symptoms were adjusted for age, sex, race, survey year, status weight and dietary behaviors including vegetable, fruit, milk, drinking and breakfast consumption. The estimates of interactive item related to suicidality were adjusted for age, sex, race, survey year, status weight, depressive symptoms and dietary behaviors including vegetable, fruit, milk, drinking and breakfast consumption. OR, odds ratio, CI: confidence interval, ***: $P<0.001$, **: $P<0.01$, *: $P<0.05$)
